# Optimal sampling and analysis methods for clinical diagnostics of vaginal microbiome

**DOI:** 10.1007/s10096-022-04545-x

**Published:** 2023-01-10

**Authors:** Katja Kero, Niina Hieta, Teemu Kallonen, Anne Ahtikoski, Hanna K. Laine, Jaana Rautava, Eveliina Munukka

**Affiliations:** 1 Department of Obstetrics and Gynecology, Turku University Hospital, University of Turku, Turku, Finland; 2 Department of Dermatology, Turku University Hospital, University of Turku, Turku, Finland; 3 Research Center for Cancer, Infections and Immunity, Institute of Biomedicine, University of Turku, Turku, Finland; 4 Department of Clinical Microbiology, Turku University Hospital, Turku, Finland; 5 Institute of Biomedicine, Microbiome Biobank, University of Turku, Turku, Finland; 6 Department of Pathology, Turku University Hospital, University of Turku, Turku, Finland; 7 Department of Oral Pathology and Oral Radiology, Institute of Dentistry, Faculty of Medicine, University of Turku, Turku, Finland; 8 Department of Oral and Maxillofacial Diseases, Faculty of Medicine, University of Helsinki and Helsinki University Hospital, Haartmaninkatu 1, 00290 ClinicumHelsinki, Finland; 9 Department of Pathology, Medicum, Faculty of Medicine, University of Helsinki and HUS Diagnostic Center, HUSLAB, Helsinki University Hospital, Helsinki, Finland; 10 Biocodex Nordics, Metsänneidonkuja 8, Espoo, Finland

**Keywords:** Vaginal microbiome, Vaginal microbiota, Microbial diagnostics sampling, Next-generation sequencing, Bacterial 16S rRNA gene

## Abstract

Next-generation sequencing-based microbiological analysis is a complex way to profile vaginal microbiome samples since each step affects the results gained. Methodologies for sample collection lack golden standards. We compared Puritan DNA/RNA swab (PS) and Copan FLOQ swab (CS) and provided consistent and reliable microbiome profiles analyzed by 16S rRNA gene sequencing. We collected two consecutive vaginal samples utilizing PS with room temperature storing and CS with instant freezing from 26 women. Variable region 4 of bacterial 16S rRNA gene was amplified with single PCR by custom-designed dual-indexed primers and sequenced with Illumina MiSeq system. Read quality control, operational taxonomic unit tables, and alpha and beta diversities analysis were performed, and community richness, diversity, and evenness were evaluated and compared between the two samplings and tests. Nineteen sample pairs produced detectable, intact DNA during the extraction protocol and/or further microbial profiles. Alpha bacterial diversity indices were independent on the collection protocol. No significant statistical differences were found in the measured beta diversity metrics between the collection methods. Of the women, 43% had *Lactobacillus*-dominated vaginal microbiome profile despite of collection method. Previously reported important vaginal microbiome phyla *Actinobacteria, Bacteroidetes, Firmicutes, Fusobacteria,* and *Proteobacteria* were present in the sample set although their relative abundances varied among individuals. PS and CS enable constant vaginal microbiota sampling. The PS method with no need for instant freezing is suitable for on-site collections at clinics. Furthermore, it seems to be possible to take two samples instead of one with constant microbiological results.

## Background

Vaginal microbiota plays a crucial role in women’s reproductive and sexual health. In the vagina, there is an ingeniously orchestrated communication between the microbes and the host providing vital defense for the host [1–5]. Enormous inter-individual variability of vaginal microbiota arises from various intrinsic and external factors such as genetics, age, diet, medications, hygiene level, and habits [1, 5]. Vaginal microbial profiles of low overall microbial diversity, dominated by certain lactobacilli species such as *Lactobacillus crispatus,* are currently considered as an example of a healthy vaginal microbiota [1, 2, 5].

Lactobacilli protect women from pathogenic microbes. The development of imbalanced microbiota composition leads to a pathological condition called dysbiosis, a state which has been linked to various disorders and diseases typical for the urogenital tract [6–10]. For example, anaerobic bacteria such as *Gardnerella, Atopobium, and Prevotella* spp. are shown to dominate microbiota in bacterial vaginosis and increase the risk of vaginal and urogenital infections as well as various sexually transmitted infections (STI) [9–13]. Recent accumulating evidence has linked unhealthy, unbalanced vaginal microbiota even to poor perinatal outcomes such as miscarriage and preterm birth, and also to severe STIs and even increased risk of cervical cancer [8, 14–16].

Conventionally, vaginal microbiota has been analyzed by light microscope with direct staining of pap smear or by traditional cultivation methods. Today, next-generation sequencing (NGS) is the method of choice for analyzing clinical human microbiota samples [1, 10, 17, 18]. Thus, the current era of microbiological research is characterized by its reliance on large data sets of nucleotide sequences and bioinformatics [17, 18]. NGS-based molecular analysis methods have increased our knowledge of the detailed vaginal microbial community composition and have provided a broader view of the microbial factors that influence the health of this rather complex vaginal ecosystem [1, 10]. Utilization of NGS as a tool in clinical diagnostics and treatment is currently under heady investigation [10, 18–20].

However, the NGS-based analysis is still quite a challenging and complex way to profile human microbiota samples since each step from sample collection to bioinformatics and final statistics affect the final results [17–21]. Further, this research field still lacks the so-called golden standards and the variety of utilized methods for example in sample collection and DNA extraction combined with diverse reporting practices make replication of studies and assessing their quality challenging [17, 22–24]. Thus, it is of outmost importance to enhance and optimize the sampling and NGS analysis procedures. These optimized procedures will improve the diagnostics, treatment, and prevention of dysbiosis and infections affecting women’s health [18, 24].

The goal of this study was to compare two vaginal microbiota sampling techniques, namely Puritan DNA/RNA swab (PS) and Copan FLOQ swab (CS), and their possible effect on the subsequent NGS analysis. First, our goal was to study a possibility of taking two consecutive samples instead of one without risking the microbiological results. The second goal was to compare two preserving methods (shield fluid reagent and dry ice) of the samples during transportation to the laboratory. The gain of several samples instead of one and the ability to work without ice and cold chain in the clinical setting would greatly benefit microbiota studies.

## Material and methods

This study is a pilot project of the EMMI study (vaginal and oral microbiota study). EMMI study is conducted at the Departments of Gynecology and Obstetrics and Dermatology of the Turku University Hospital and Institute of Dentistry, University of Turku, Turku, Finland. EMMI study has been reviewed by the Ethics Committee of the Hospital District of Southwest Finland (nro 97/1801/2016).

This pilot project of the EMMI study includes a population of non-pregnant women (*n* = 26, mean 39.1 years, age range 21–68). These women were referred to the Department of Gynecology and Obstetrics, Turku University Hospital because of an abnormal finding in a pap smear test for further examination by colposcopy. Written, informed consent was obtained from each of the volunteers prior to the sample collection. Further, they filled a questionnaire of their demographic characteristics.

### Vaginal sampling and DNA extraction

**Fig. 1:**
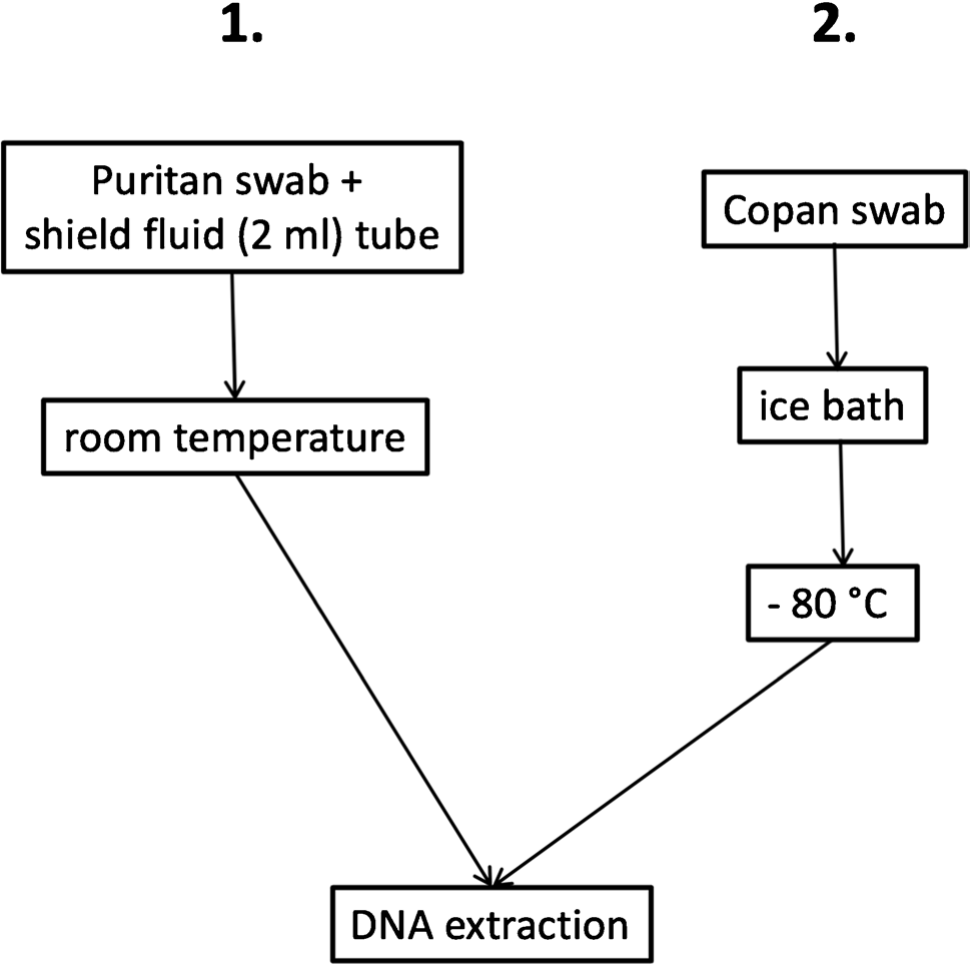
Vaginal sample collection and wet lab workflow. Consecutive two samples were taken from the identical vaginal site. Firstly, sterile Puritan DNA/RNA swab (Puritan Medical products, Guilford, ME, USA) was rotated in vagina to collect discharge and placed in a 2-mL shield fluid collection tube (Zymo Research, Irvine, Canada). The tube was kept at room temperature. Secondly, a Copan FLOQ swab (CopanDiagnostics, Murrieta, CA, USA) was rotated in the vagina and transferred immediately on dry ice. Samples were transferred after the collection point to the laboratory of Microbiome Biobank, Turku, Finland. Puritan Shield fluid samples were stored at room temperature and Copan FLOQ swabs at − 80 °C until the DNA extraction

Vaginal study samples were collected on-site, at the beginning of the clinical visit prior to any other investigations and procedures by a specialist in gynecology. Two consecutive swabs were collected and preserved in different sampling tubes namely the Puritan shield fluid tube (PS) and Copan FLOQ tube (CS) from each individual. The sampling flow chart representing the two collection tubes is presented in Fig. 1. Samples were transferred after the collection to the laboratory of Microbiome Biobank, Turku, Finland. PS samples were stored at room temperature and CS at − 80 °C until the DNA extraction, which was made within two weeks according to the manufacturer’s protocol.

Bacterial DNA was extracted with Hain Viral N/A Extraction Kit and GenoXtract machine (Hain Lifescience GmbH, Nehren, Germany) from the 200 μl aliquot of PS. The biological content collected by CS was dissolved in 200 μl of Puritan Shield fluid prior to the extraction. DNA concentrations were measured with Qubit dsDNA HS Assay Kit and Qubit 2.0 fluorometer (Life Technologies, Carlsbad, USA). Extracted DNAs were stored at − 80 °C prior to the NGS approach.

### Next-generation sequencing analysis

Nineteen of the original 26 sample pairs produced detectable DNA and/or microbial profiles with sequencing. Variable region 4 (V4) of bacterial 16S rRNA gene was amplified with single PCR by custom-designed dual-indexed primers and sequenced with Illumina MiSeq (Illumina, San Diego, California, USA) system as previously described [25]. Briefly, the KAPA HiFi PCR kit (KAPA Biosystems, Massachusetts, USA) with in-house generated primers was utilized in amplification. Forward and reverse primer sequences were 5′-AATGATACGGCGACCACCGAGATCTACAC-i5-TATGGTAATT-GT-GTGCCAGCMGCCGCGGTAA-3′ and 5′-CAAGCAGAAGACGGCATACGAGAT -i7- AGTCAGTCAG-GC-GGACTACHVGGGTWTCTAAT-3′, respectively, where i5 and i7 represent the sample-specific index sequences.

The PCR products were purified with Agencourt AMPure XPMagnetic beads (BeckmanCoulter, Inc., USA) on DynaMag™-96 magnetic plate (Life Technologies, USA). The PCR product length and DNA integrity were checked with TapeStation (Agilent Technologies Inc., USA), and the final DNA concentrations of the purified products were measured with Qubit 2.0 dsDNA HS assay kit (Life Technologies, USA). The products were then mixed in equal concentrations to generate a 4 nM library pool, which was denatured, diluted into a final concentration of 4 pM, and spiked with 25% denatured PhiX control (Illumina, USA) for sequencing. Sequencing was done with 2 × 250 bp paired-end reads on the MiSeq system (Illumina, USA), using MiSeq v3 reagent kit (Illumina, USA). Raw reads across the samples sequenced with the Illumina MiSeq 250 bp paired-end sequencing were used as input for the data analysis.

### Data processing and statistical methods

Read quality control, operational taxonomic unit (OTU) tables, alpha, and beta diversities analysis were performed with CLC Genomics Workbench v. 20 Microbial Genomics module (QIAGEN Digital Insights, Aarhus, Denmark). Raw sequences were assigned to operational taxonomic units (OTU) according to the CLC Microbial genomics module workflow. Quality and ambiguous trims were performed with default settings, and the minimum number of nucleotides was set to 150. The minimum rarefraction level was 5264. SILVA 16S v132 preclustered at 97% identity was used as the reference database [26, 27]. Alpha diversity measures, namely Chao1 index, Shannon index, and number of observed species were calculated to evaluate community richness, diversity, and evenness. The beta diversity measure, Bray–Curtis dissimilarity, was calculated and the PERMANOVA test with 99,999 permutations was used to calculate the *p*-values.

Kruskal–Wallis H test was used to assess whether the values depend on the group they belong to in Chao1 and Shannon indices.

## Results

### The effect of the sample collection method on the DNA yields

There was a significant difference in the DNA gain (nanogram/microliter of vaginal sample) between the two evaluated sampling methods, CS and PS: 3.2 ± 4.0 vs. 15.6 ± 14.6 ng/µl (*p* < 0.001). Four CS samples contained too low DNA gain or quality and were excluded from the MiSeq analysis. Thus, the DNA quantity when using dry CS and immediate freezing was lower in all the collected sample pairs than in PS.

### Overall sequencing output and microbial profiles

**Fig. 2:**
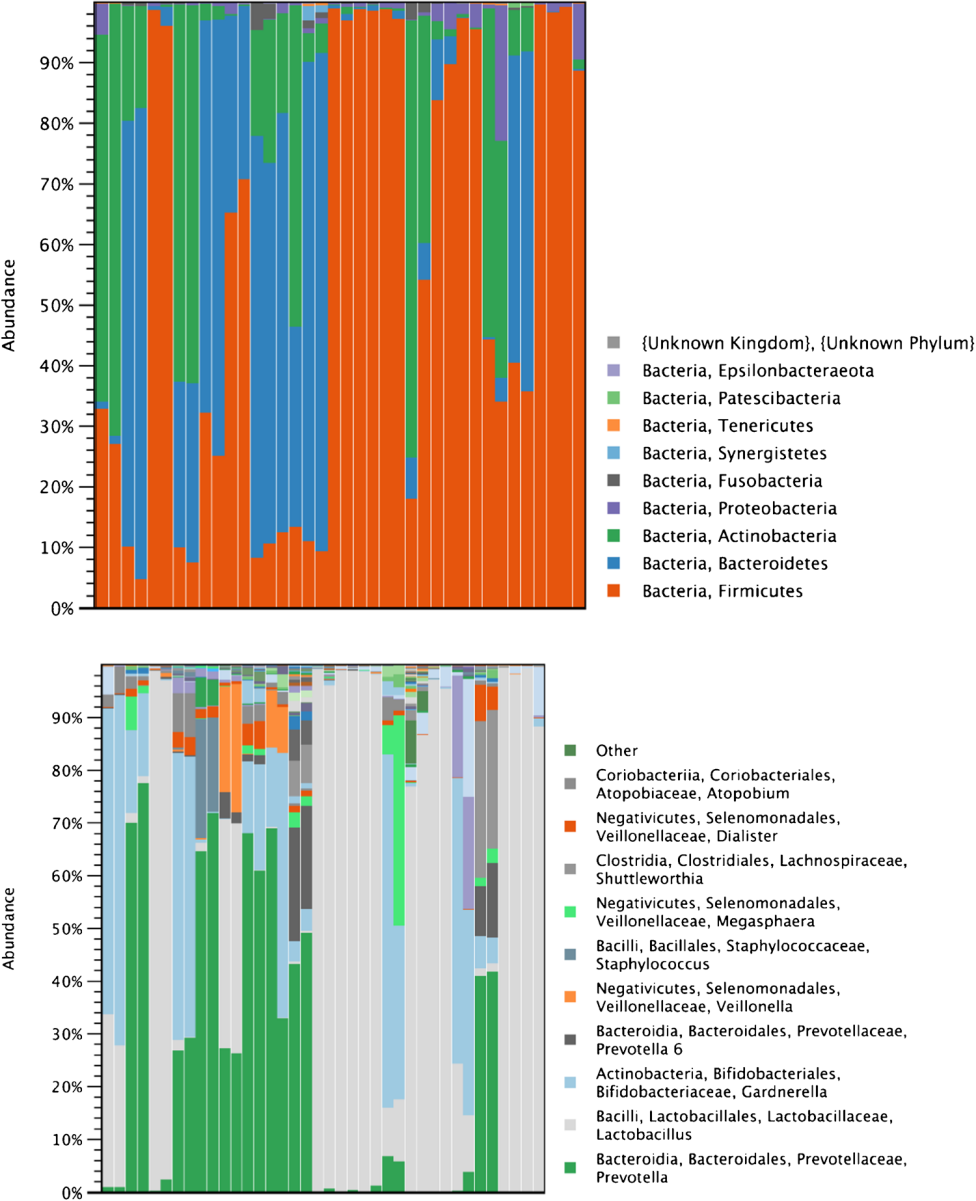
Stacked barplots representing relative abundance of bacteria on consecutive vaginal microbiome samples in phylum (upper chart) and genus level (lower chart). All the gained 16S rRNA taxonomical profiles represented bacterial taxa that are characteristic of vaginal microbiota despite the collection method

Three samples did not provide quality results and were excluded. A total of 18, 327, 359 reads from the Illumina MiSeq platform of the 19 samples were trimmed for further analyses**.** All sequenced samples produced detectable microbial profiles. However, there was some variation between the sequence counts between the different individuals (Fig. 2). Despite the collection method, all gained 16S rRNA taxonomical profiles represented bacterial taxa that are characteristic of vaginal microbiota (Fig. 2).

### Vaginal microbiota

Members of all the previously reported important phyla *Actinobacteria, Bacteroidetes, Firmicutes, Fusobacteria,* and *Proteobacteria*, were present in the sample sets although their relative abundances varied markedly from person to person (Fig. 2 upper chart). Altogether, 8/19 (42%) of the women had *Lactobacillus*-dominated vaginal microbiome despite of collection method (Fig. 2 lower chart).

### Diversity indices

**Fig. 3:**
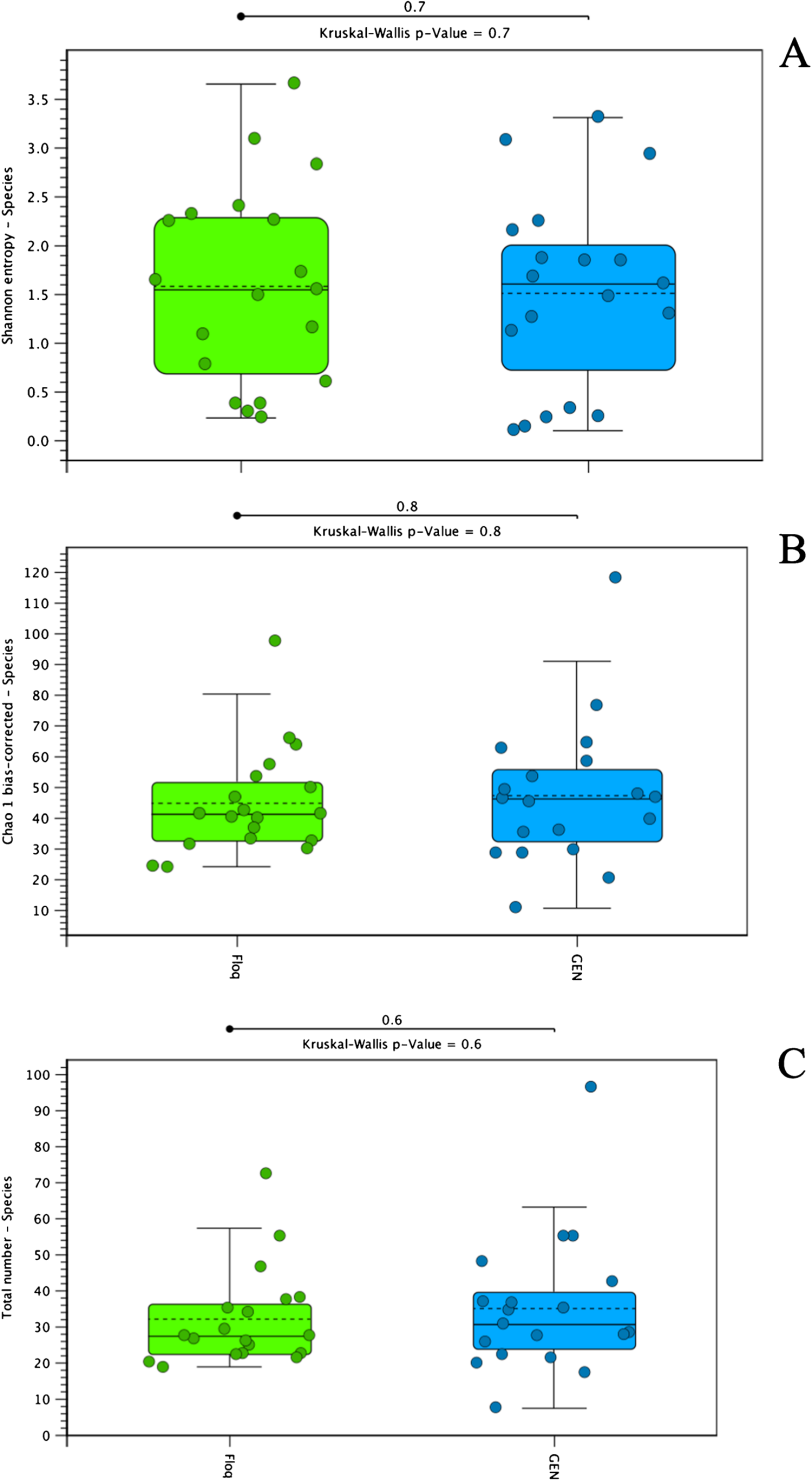
Observed alpha bacterial diversity indices, represented as **A** Shannon index (*p* = 0.7), **B** Chao1 index (*p* = 0.8), and **C** the observed number of species (*p* = 0.6), was not dependent on the collection protocol

**Fig. 4:**
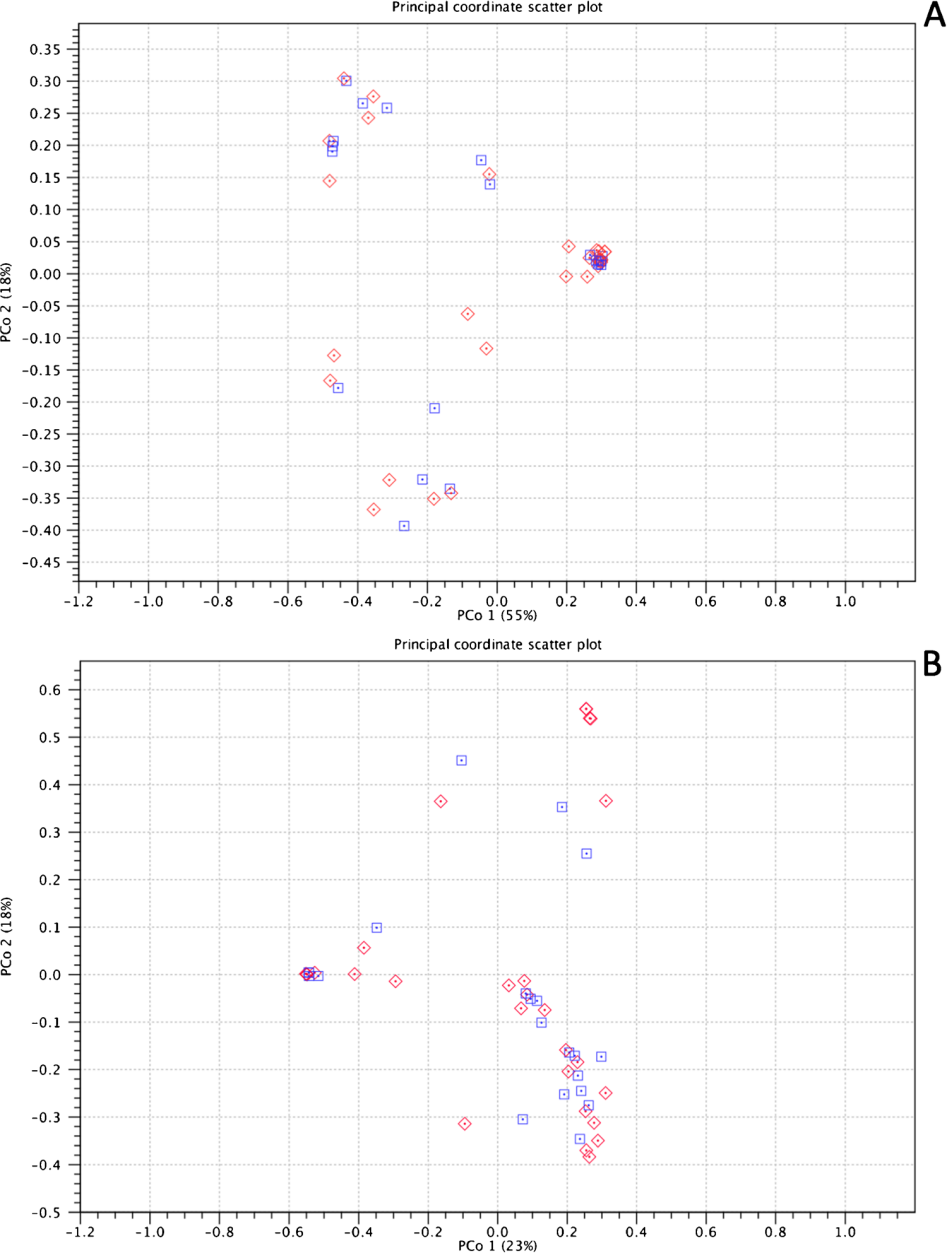
Neither **A** weighted UniFrac nor **B** Bray Curtis β-diversity metrices were significantly different between the collection methods (*p* > 0.05, for both) indicating that both CS and PS are acceptable means of sampling. Blue = CS, red = PS

The overall microbial profiles of vaginal samples were further described by utilizing various diversity measures. Observed alpha bacterial diversity indices, represented as Shannon index, Chao1 index, and the observed number of species, were not dependent on the collection protocol (*P* > 0.05 for all, Fig. 3A-C). In addition, no significant statistical differences were found in any of the measured β-diversity metrics between the collection methods (*p* > 0.05 for all, Fig. 4A, B) indicating that both CS and PS are acceptable means of sampling.

## Discussion

Microbial diseases and disorders affecting female genital health have become an increased epidemiological and clinical challenge and also have a social and psychological influence. Vaginal microbial composition is a potential future target for clinical diagnostics [10, 20, 24, 28–30]. However, the development of any diagnostic test in clinical microbiology requires straightforward and trustworthy sample-collection methods.

In the present study, DNA gain was different between the two evaluated sampling methods as reported also in earlier methodological studies [23]. This may reflect issues with the sampling method such as the structure of the swab. However, since the gain reduced with the latter utilized method, the possibility is that the first sampling had a better yield simply because it was performed first. However, this reduced gain was able to provide similar results on the microbiota. Furthermore, both sample collection methods produced 16S rRNA taxonomical profiles that were similarly distinguishable between *Lactobacillus*-dominant versus mixed microbiota. Bacterial diversity was not dependent on the collection protocol. Therefore, it seems safe to collect two samples at the same visit from the vaginal site with no influence on microbiota results. The gain of two vaginal samples instead of one will increase the possibilities for microbiota research.

Based on molecular studies, there are an estimated 10^12^ − 10^13^ fungi compared to 10^13^ − 10^14^ bacteria in the human microbiota, across the gastrointestinal tract, oral cavity, vaginal mucosa, and skin [31]. According to previous studies as mentioned above, in cohort representative of a normal healthy female population, the vaginal microbiome has shown five subgroups where four of the groups have contained lactobacillus dominated and one group non-lactobacillus dominated microbiome [1, 10, 23, 32, 33]. In our study, 42% of the women had *Lactobacillus*-dominated vaginal microbiome. Our primer set included also V4 level primers [25] and thus, we were able to show the presence of *Gardnerella* spp. (Fig. 3). The presence of *Gardnerella vaginalis* and an assortment of other, typically anaerobic species is indicative of dysbiosis and prevails, e.g., in bacterial vaginosis [9, 34, 35]. However, this was expected since our cohort consisted of women referred to colposcopy due to an abnormal pap smear finding, thus not representing a normal population. An abnormal pap smear is linked with human papillomavirus (HPV) positivity and further dysbiosis [8].

As a strength of the current study, with V4-targeted 16S amplicon sequencing analysis, it is possible to get an overview of the bacterial community composition of a clinical sample and further, in clinical studies identify profile-level differences between the groups and variation within groups [18, 24]. Research work is time and money consuming. In the current study, we have simplified the sampling method and succeeded in taking two consecutive samples and working on room-temperature material in the clinical setting. This increases the gain of research material with fewer appointments for sampling and the lack of ice and cold chain opens new and more distant possibilities for research. However, our study also has several limitations. This study was designed only to methodologically compare the microbial results of two consecutive vaginal samplings focusing on two different sampling methods. In addition, the design of this pilot study with a small number of participants did not allow randomization due to clinical practice and only one researcher.

As a conclusion, we demonstrate that it was safe to collect two consecutive samples from the same vaginal site with minimal influence on microbiota results. PS and CS enabled constant vaginal microbiota sampling without differences between the two sampling methods. In addition, shield fluid reagent allows the transportation of microbiota samples at room temperature as it preserves the integrity of genetic material present in samples at ambient temperatures enabling it to be used in NGS analysis. A redundant cold chain simplifies collection and research in more distant locations from research utilities.

## Data Availability

The datasets generated during and analyzed during the current study are not publicly available but are available from the corresponding author on reasonable request.
